# Brain Connectivity Exposed by Anisotropic X-ray Dark-field Tomography

**DOI:** 10.1038/s41598-018-32023-y

**Published:** 2018-09-25

**Authors:** Matthias Wieczorek, Florian Schaff, Christoph Jud, Daniela Pfeiffer, Franz Pfeiffer, Tobias Lasser

**Affiliations:** 1 Computer Aided Medical Procedures, Technical University of Munich, 85748 Garching, Germany; 2 Chair of Biomedical Physics, Department of Physics and Munich School of BioEngineering, Technical University of Munich, 85748 Garching, Germany; 3 Department of Diagnostic and Interventional Radiology, Klinikum rechts der Isar, Technical University of Munich, 81675 München, Germany; 4 Institute for Advanced Study, Technical University of Munich, 85748 Garching, Germany

## Abstract

To understand the interaction of different parts of the human brain it is essential to know how they are connected. Such connections are predominantly related to the brain’s white matter, which forms the neuronal pathways, the axons. These axons, also referred to as nerve fibers, have a size on the micrometer scale and are therefore too small to be imaged by standard X-ray systems. In this paper, we use a grating interferometer and a method based on Anisotropic X-ray Dark-field Tomography (AXDT) with the goal to generate a three-dimensional tomographic reconstruction of these functional structures. A first preclinical survey shows that we successfully reconstruct the orientations of the brain fibers connectivity with our approach.

## Introduction

From an abstract point of view, the central nervous system (CNS), and especially the human brain, consists of neurons, which are connected by axons/nerve fibers. The latter transmit signals between neurons and are thus a main ingredient to build the neural network. In order to understand how communication within the brain is carried out, it is a key interest to understand this connectivity. Visualization of the structural connectivity of the brain is of major interest in biomedical imaging. Research activities of the embryonic development of the brain need information on morphological connections of fibers to understand the functional communication of different cerebral areas^1–3^. In a clinical setting, illustration of white matter tracts is essential for planning of neurosurgical procedures^4^. Especially in patients with brain tumors, which are located close to fundamental cerebral areas like the motor tract, visualization of the white matter tracts is crucial for planning of tumor resection and clinical outcome^5^.

Currently, the gold standard for imaging these fibers is diffusion tensor imaging (DTI)^6^ and related but improved methods such as q-ball imaging^7^. These methods are based on diffusion magnetic resonance imaging (Diffusion-MRI) which provides a measure of the diffusion process of water molecules at sub-voxel level in each location within the human brain. These diffusion-based methods have provided huge advances in the understanding of the human brain. For example, they have been applied for detection of brain ischemia^8^, investigation of autism^9^, or neurodegenerative pathologies such as the Huntington’s disease^10^ or schizophrenia^11,12^. A review on the applications of DTI is provided in^13^.

X-ray computed tomography (CT), on the other hand, is typically superior to MRI when it comes to imaging speed, resolution, and availability. However, current clinical X-ray CT machines lack the necessary resolution and functional imaging capability to recover individual nerve fibers of sizes on the micrometer scale (c.f.)^14,15^. Dedicated (non-medical) micro- and nano-CT devices are in principle capable of resolving structures on the nm and μm length-scale, but suffer from a very limited field of view, i.e. in the μm, mm regime. Another alternative to get access to the fiber orientation is provided by recently developed methods based on (ultra-) small-angle X-ray scattering (SAXS)^16–18^. Unfortunately, these methods require very long scanning times and even more crucially, highly brilliant synchrotron radiation, making them inaccessible in the medical context.

## Anisotropic X-ray Dark-field CT Imaging

**Figure 1:**
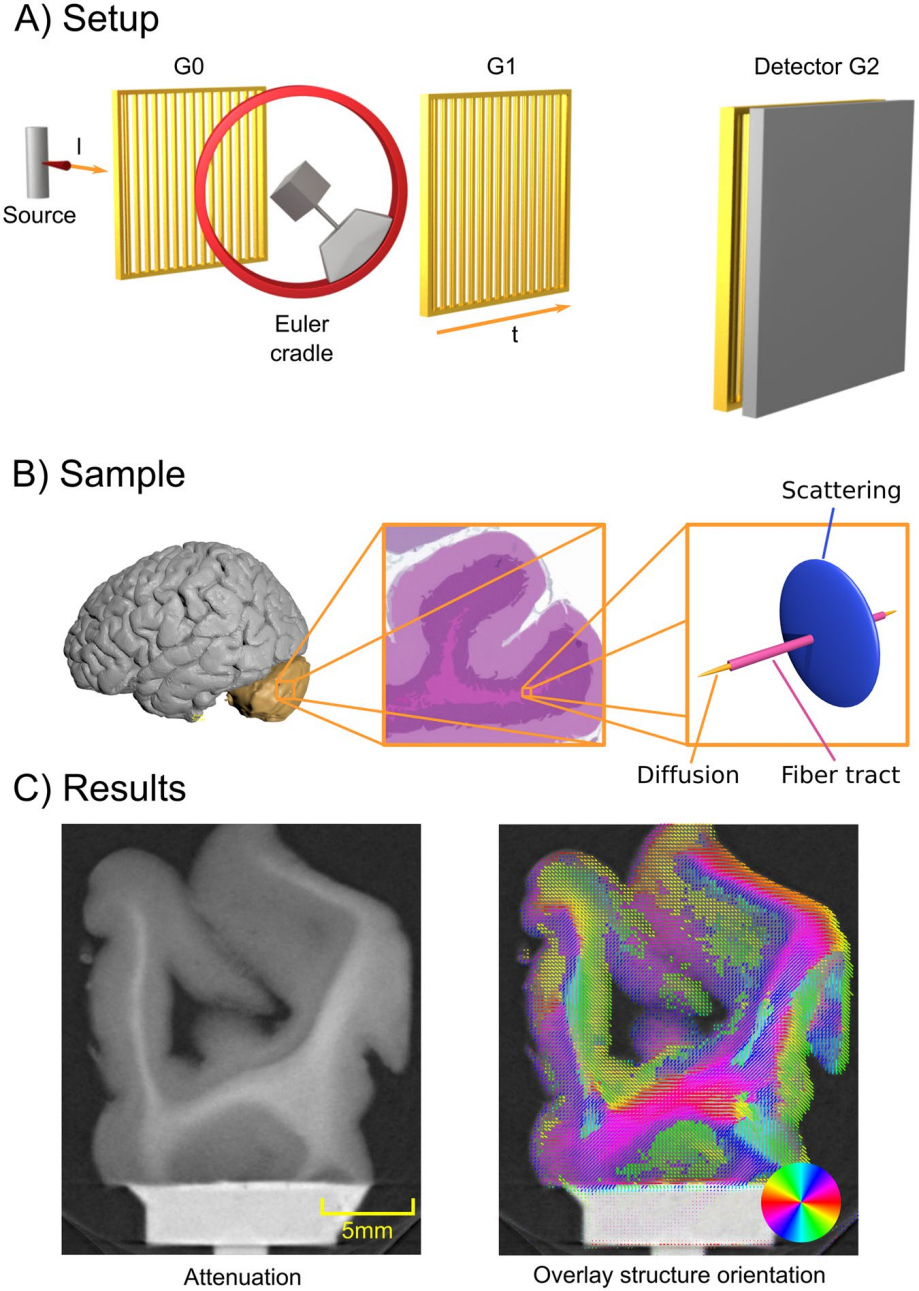
A general setup overview is shown in (**A**). A standard X-ray imaging setup with a source and a detector is augmented with two absorption gratings G0 and G2 and one phase grating G1. Additionally, a Euler cradle is used to rotate the sample in a fully three-dimensional manner. Additionally, the grating orientation *t* and one X-ray direction *l* are illustrated. In B) we illustrate the location of the cerebellum within the human brain (left)^32^. In the middle of (**B**) we show a schematic histology image of the cerebellum with H&E stain. The center region of the histology image shows the fiber tracts located in the white matter of human brain tissue, aligning with it. Finally, on the right side of (**C**) we sketch the relationship of the fiber tracts, the diffusion MRI and the assumed scattering signal. In (**C**) we show a slice of conventional attenuation based CT on the left, and on the right an overlay of the additional directional information obtained by the proposed method in this paper.

In this paper, we present a method based on grating-based X-ray imaging, namely dark-field imaging^19^, which overcomes the limitations mentioned above. It provides structural information on the brain fiber orientation, but is at the same time capable of being implemented with a large field of view (similar to clinical CT). In Fig. 1A) we illustrate such a grating-based X-ray imaging setup, which consists of a standard X-ray CT setup with three additional gratings, enabling the simultaneous extraction of the additional phase-contrast^20^ and dark-field-contrast^19^ signals. It has been shown that, in particular, the dark-field signal is correlated to (ultra-) small angle X-ray scattering^21^ of the aligned micro- and nano-structures in the sample. For dark-field contrast grating-based setups, the grating bars mainly pick up scattering occurring orthogonally to the grating bars, meaning that the signal is strongest if a microstructure aligns with the grating bars^22,23^. Consequently, for highly structured specimens, the dark-field signal constitutes an anisotropic quantity. This observation lead to the method of X-ray vector radiography^24^.

So far, the concept of dark-field imaging has been successfully used for various applications, including lung imaging^25^, musculoskeletal imaging^26^, materials testing^27^, or imaging of dental samples^28^. Aiming at tomographic reconstruction of this anisotropic signal, we recently proposed a method called Anisotropic X-ray Dark-Field Tomography (AXDT) in^29^, based on the previous work performed by Malecki *et al*.^30^ and Vogel *et al*.^31^. In order to properly sample this anisotropic signal, the grating-based setup is additionally augmented with a Euler cradle, enabling fully three-dimensional rotation of the sample. AXDT allows the tomographic reconstruction of the scattering profile in each location of the specimen, which in turn enables the imaging of nerve fibers for the very first time.

## Scattering and Diffusion in the Human Brain

In order to show the potential of AXDT specifically for brain connectivity applications in a first proof-of-principle feasibility study, we investigated a sample from the region of the cerebellum in a human brain. (Fig. 1B)^32^ shows the location of the cerebellum on the left side. In the middle, a schematic illustration of a histology slice of a human cerebellum depicts the gray matter and white matter. To the right, a sketch of the relation of diffusion-MRI (gold), fiber tracts (purple) and scattering on the right side (blue), is shown. While the diffusion process (gold) aligns with the fiber tract (purple), the scattering profile (blue) is supposed to be strongest orthogonal to the aligned structures. The cerebellum can be divided into grey matter and white matter. In Fig. 1B) center we show schematic illustration of a histology slice of a cerebellum with hematoxylin and eosin (H&E) stain leading to a violet/red coloring. The white matter mainly consists of axons, which build a junction between different areas within the central nervous system. Those axons are arranged in long bundles, leading to a mainly aligned/parallel arrangement of the fibers to each other. The grey matter contains multiple different nerve cells, which can be subdivided in three different layers: stratum moleculare, stratum purkinjense, and stratum granulosum, respectively. Parallel fibers represent the axons of the granule cells and build up connections between the different cells within the grey matter.

**Figure 2:**
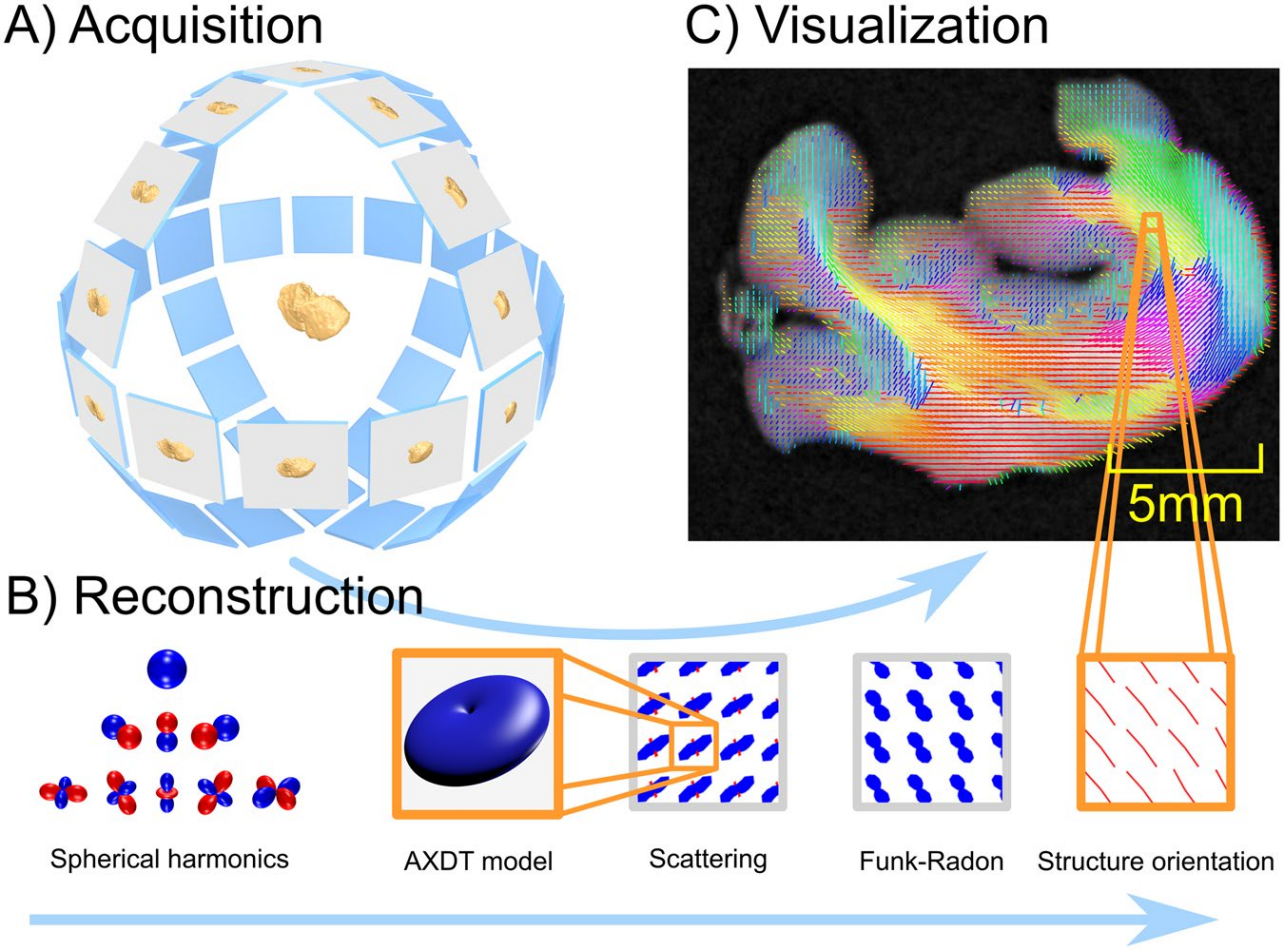
In (**A**)^32^ we illustrate the acquisition scheme, which in contrast to standard CT, requires fully three-dimensional rotation of the sample instead of one single axis of rotation. Subfigure (**B**) illustrates the reconstruction pipeline. Firstly, the scattering in each position of the specimen is modeled via spherical harmonics. Secondly, this scattering is reconstructed using tomographic reconstruction. Thirdly, the scattering information is transformed via the Funk-Radon and local maxima are extracted to obtain the orientations of the scattering profiles. In (**C**) we sketch the embedding of these reconstructed fiber orientation in our final result.

In Fig. 2 we show an overview of our method. The method basically consists of three parts: First acquisition, second tomographic reconstruction, and third the final fiber orientation extraction.

## Acquisition

As illustrated in Fig. 1A), a Talbot-Lau interferometer setup is used. In order to sufficiently sample the scattering signal, the sample needs to be rotated in a fully three-dimensional manner. This also implies that a standard tomographic axis as used in standard X-ray CT is not sufficient. In order to sample the specimen more freely, a Euler cradle is added to the setup. This device allows to freely rotate the object in a fully three-dimensional manner (compare Fig. 2A)^32^. Optimal acquisition protocols for this novel imaging modality is an ongoing field of research (c.f ^33^).

## Anisotropic X-ray Dark-field Tomography (AXDT)

For tomographic reconstruction of the spherical scattering distribution we use the recently proposed method of AXDT (compare Fig. 2B)). Within this method, the scattering is described as a 3D-field of spherical functions, mapping a specific scattering direction to a scattering strength. As proposed in^29^ we use real-valued spherical harmonics in order to express these spherical functions at each location inside the reconstruction volume. Based on this we introduced a forward model for the anisotropic dark-field measurements based on the resulting spherical harmonics coefficients. This enables the tomographic reconstruction of the scattering profile in each location of the specimen with respect to spherical harmonics.

## Fiber Tract Extraction

We already illustrated that the effect of scattering is strongest orthogonal to the microstructure/fiber in Fig. 1B). Therefore, we are interested in the direction orthogonal to scattering occurring on plate-like shapes. For this purpose, we^34^ recently proposed to utilize the Funk-Radon transform^35^, which transforms spherical functions to the integral values of great circles on the unit sphere. Thus, strong scattering on a plate-like shape will be translated to the accumulated value in orthogonal direction, which is assumed to be the direction of the fiber. Compare Fig. 2B), which shows the relation between the reconstructed scattering profiles, their Funk-Radon and the extracted structure orientations.

## Experiments and Results

All methods were performed in accordance with the relevant guidelines and regulations. For our experiments, a piece of a human cerebellum was used. The sample was excised at the Institute of Forensic Medicine (Ludwig Maximilian Universität München, Germany) and is part of the ethics applicant 319/13, which was approved by the ethics commission of the Faculty of Medicine of the Technische Universität München. The review board waived the need for consent as this sample was excised for forensics.

**Figure 3:**
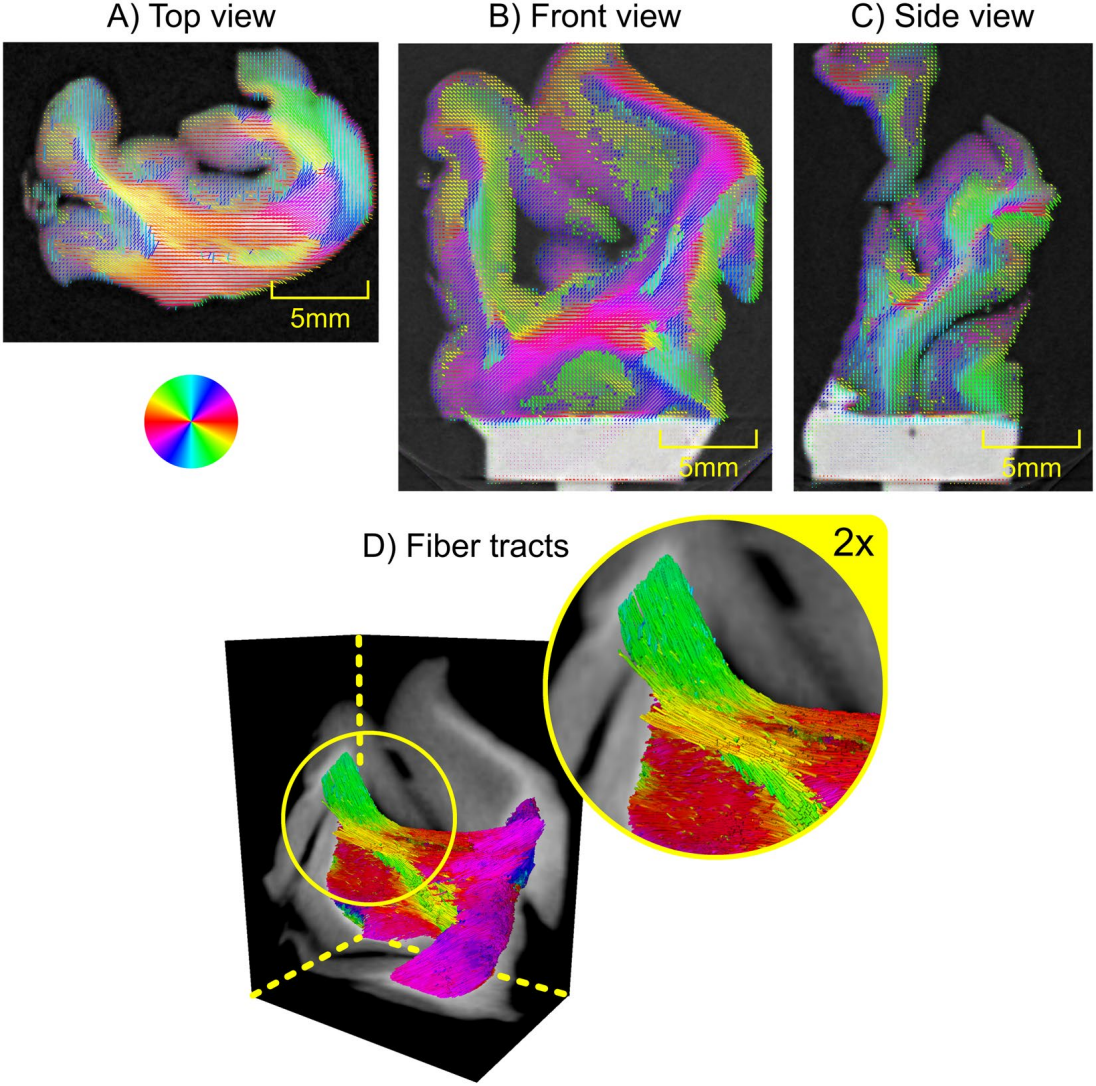
(**A**–**C**) Three slices of the attenuation X-ray CT overlaid with the fiber tract orientations as produced by our approach of Anisotropic X-ray Dark-field Tomography (AXDT). The coloring shows the in-plane orientation according to the color-wheel at the top left. In (**D**) we display three cross-sections of the 3D attenuation X-ray CT overlaid with the fiber tracts within the white matter as computed with a streamline method. The coloring was chosen based on the orientation of the tracts projected onto the plane orthogonally to the viewing direction according to the color-wheel.

The sample was critical-point-dried prior to any measurements. This allowed for a simplified sample mounting and specimen stability over the rather long measurement times of this proof-of-principle study, but in general no special sample preparation is required for this method. The sample was then measured and reconstructed using AXDT. The exact parameters are discussed in the Supplemental Material. In Fig. 3 we display the three center slices of the reconstructed data. The structure orientations (a vector field) extracted from the AXDT reconstruction are overlaid over a three-dimensional rendering of the corresponding attenuation X-ray CT. The structure orientations have been colored based on their in-plane orientation according to the given color-wheel. Additionally, their length corresponds to the accumulated scattering orthogonal to their orientation, i.e. the value of the Funk-Radon transform in that direction. In Fig. 3D) we additionally visualize the fiber tracts within the white matter, as computed via a streamline method tracking fibers along the vector field of the orientations in 3D.

In the non-white matter regions, we predominantly find less strong and less structured scattering, which fits the circumstance that this region, in addition to axons, contains stellate and basket cells. Within the white matter though, we find the strongest scattering signal and strongly structured orientations. This fits the knowledge from medicine e.g. from histology images such as illustrated in Fig. 1.

Besides the complementary information, with an effective voxel size of 0.5 mm for this study we are already below the resolution of current Diffusion MRI, which is typically in the mm regime. In fact, since we are binning our data by a factor of 4 for computational speed, voxel sizes of 0.125 mm are immediately possible.

## Conclusion

In this paper we presented a method for imaging nerve fibers connectivity across macroscopic samples of human brain tissue using AXDT. We presented first preclinical results, which indicate the successful tomographic reconstruction of the directions of nerve fibers in the white matter of the cerebellum. This is particularly interesting, as these fibers are much smaller than the employed detector resolution. Further, as this method poses a complementary imaging method to both attenuation-based X-ray imaging and Diffusion MRI, we are positive that this method will provide additional insight into human anatomy. As any functionality within the human CNS is built upon the connectivity based on nerve fiber tracts, this insight can provide additional insight in the basic functionality, for planning neurosurgeries, and for investigation of CNS related diseases.

## Electronic supplementary material

Supplementary Materials

## Data Availability

The data that support the findings of this study are available from the corresponding author upon reasonable request. The software code used for the AXDT reconstruction as well as the code used for the fibre extraction and their visualization can be accessed from the corresponding author upon request.

## References

[1] Watts R, Liston C, Niogi S, Ulu AM (2003). Fiber tracking using magnetic resonance diffu- sion tensor imaging and its applications to human brain development. Mental Retardation and Developmental Disabilities Research Reviews.

[2] Dubois J, Hertz-Pannier L, Dehaene-Lambertz G, Cointepas Y, Le Bihan D (2006). As- sessment of the early organization and maturation of infants’ cerebral white matter fiber bundles: A feasibility study using quantitative diffusion tensor imaging and tractography. NeuroImage.

[3] Yoo S-S (2005). *In Vivo* Visualization of White Matter Fiber Tracts of Preterm- and Term- Infant Brains With Diffusion Tensor Magnetic Resonance Imaging. Invest. Radiol..

[4] Nimsky C (2005). Preoperative and Intraoperative Diffusion Tensor Imaging-based Fiber Tracking in Glioma Surgery. Neurosurgery.

[5] Bello L (2008). Motor and language DTI Fiber Tracking combined with intraoperative subcortical mapping for surgical removal of gliomas. NeuroImage.

[6] Basser PJ, Mattiello J, LeBihan D (1994). MR diffusion tensor spectroscopy and imaging. Biophys. J..

[7] Tuch DS (2004). Q-ball imaging. Magnetic Resonance in Medicine.

[8] Le Bihan D (2001). Diffusion tensor imaging: concepts and applications. J Magn Reson Imaging.

[9] Alexander AL (2007). Diffusion tensor imaging of the corpus callosum inAutism. . Neu- roImage.

[10] Rosas HD (2010). Altered white matter microstructure in the corpus callosum in Hunt- ington’s disease: Implications for cortical “disconnection”. NeuroImage.

[11] Foong J (2000). Neuropathological abnormalities of the corpus callosum in schizophrenia: a diffusion tensor imaging study. Journal of Neurology, Neurosurgery & Psychiatry.

[12] Kubicki M (2007). A review of diffusion tensor imaging studies in schizophrenia. Journal of Psychiatric Research.

[13] Assaf Y, Pasternak O (2007). Diffusion Tensor Imaging (DTI)-based White Matter Mapping in Brain Research: A Review. J Mol Neurosci.

[14] Wang H (2011). Reconstructing micrometer-scale fiber pathways in the brain: multi-contrast optical coherence tomography based tractography. NeuroImage.

[15] Jayachandra MR, Rehbein N, Herweh C, Heiland S (2008). Fiber tracking of human brain using fourth-order tensor and high angular resolution diffusion imaging. Magnetic Resonance in Medicine.

[16] Schaff, F. *et al*. Six-dimensional real and reciprocal space small-angle X-ray scattering tomography **527**, 353–356 (2015). 10.1038/nature16060 26581292

[17] Liebi M (2015). Nanostructure surveys of macroscopic specimens by small-angle scattering tensor tomography. Nature.

[18] Jensen TH (2011). Brain tumor imaging using small-angle x-ray scattering tomography. Phys. Med. Biol..

[19] Pfeiffer F (2008). Hard-X-ray dark-field imaging using a grating interferometer. Nat. Mater..

[20] Pfeiffer F, Weitkamp T, Bunk O, David C (2006). Phase retrieval and differential phase- contrast imaging with low-brilliance X-ray sources. Nat. Phys..

[21] Bech, M. *et al*. Experimental validation of image contrast correlation between ultra-small- angle X-ray scattering and grating-based dark-field imaging using a laser-driven compact X-ray source. *Photonics and Lasers in Medicine* **1** (2012).

[22] Jensen TH (2010). Directional x-ray dark-field imaging. Phys. Med. Biol..

[23] Jensen TH (2010). Directional x-ray dark-field imaging of strongly ordered systems. Phys. Rev. B.

[24] Potdevin G (2012). X-ray vector radiography for bone micro-architecture diagnostics. Phys. Med. Biol..

[25] Yaroshenko A (2013). Pulmonary Emphysema Diagnosis with a Preclinical Small-Animal X-ray Dark-Field Scatter-Contrast Scanner. Radiology.

[26] Schaff, F. *et al*. Correlation of X-Ray Vector Radiography to Bone Micro-Architecture. *Sci*. *Rep*. **4** (2014). 10.1038/srep03695 PMC3892438 24424256

[27] Revol V (2013). Laminate fibre structure characterisation of carbon fibre-reinforced poly- mers by X-ray scatter dark field imaging with a grating interferometer. NDT & E INT..

[28] Jud C (2016). Dentinal tubules revealed with X-ray tensor tomography. Dental Materials.

[29] Wieczorek M, Schaff F, Pfeiffer F, Lasser T (2016). Anisotropic X-Ray Dark-Field Tomog- raphy: A Continuous Model and its Discretization. Phys. Rev. Lett..

[30] Malecki A (2014). X-ray tensor tomography. EPL (Europhysics Letters).

[31] Vogel J (2015). Constrained X-ray tensor tomography reconstruction. Opt. Express.

[32] The brain image is created using the Brainder project with the kind permission of A. Winkler https://brainder.org, publicly available under the Creative Commons Attribution-ShareAlike 3.0 License (https://creativecommons.org/licenses/by-sa/3.0/).

[33] Sharma Y, Schaff F, Wieczorek M, Pfeiffer F, Lasser T (2017). Design of Acquisition Schemes and Setup Geometry for Anisotropic X-ray Dark-Field Tomography (AXDT). Sci. Rep..

[34] Wieczorek, M., Pfeiffer, F. & Lasser, T. Micro-structure orientation extraction for Anisotropic X-Ray Dark-Field Tomography. In *Fully3D* (2017).

[35] Funk P (1913). Über Flächen mit lauter geschlossenen geodätischen Linien. Math.Ann..

