# Supplementary material for: Brain Connectivity Exposed by Anisotropic X-ray Dark-field Tomography
Source: Sci Rep. 2018 Sep 25;8:14345. doi: 10.1038/s41598-018-32023-y (PMC6156569; doi:10.1038/s41598-018-32023-y)
Supplement: Supplementary file 1 — Supplementary Materials [file 41598_2018_32023_MOESM1_ESM.pdf]

Supplementary Materials for

Brain Connectivity Exposed by Anisotropic X-ray Dark-field Tomography

Matthias Wieczorek, Florian Schaff, Christoph Jud, Daniela Pfeiffer, Franz Pfeiffer, and Tobias Lasser
correspondence to: lasser@cs.tum.edu

Supplementary Materials:

Materials and Methods: All methods were performed in accordance with the relevant guidelines and regulations. For our experiments, a piece of a human cerebellum was used. The sample was excised at the Institute of Forensic Medicine (Ludwig Maximilian Universität München, Germany) and is part of the ethics applicant 319/13, which was approved by the ethics commission of the Faculty of Medicine of the Technische Universität München. The review board waived the need for consent as this sample was excised for forensics.

The sample was dried with a critical point drying method. In detail, the sample was originally embedded in a formalin solution, which was replaced by acetone step by step. Afterwards, the sample was inserted into the critical point dryer, sealed and the temperature was reduced to approximately 7 °C. The sample was then flushed with CO_2 ten times. Following the drying process, the remaining gas was removed and the temperature was raised to approximately 32 °C.

For our experiments, we used a symmetrical Talbot-Lau grating interferometer setup with an inter-grating distance of 920 mm as proposed in (1) (see fig. 1 A)). Two gold-based attenuation gratings with a period of 10 µm were used as G0 and G2. The nickel-based G1 phase-grating with a period 5µm was designed to provide a π/2-phase-shift at a photon energy of 45 kVp. Accordingly, the interferometer was run at the first fractional Talbot distance. X-rays were generated with a tungsten-target X-ray WorX 160-SE microfocus X-ray tube, operated at 60kVp and anode current of 1.66 mA. Images were recorded with a Varian PaxScan 2520 DX flat-panel detector with a CsI scintillator screen. An area of 800×800 pixels with an isotropic pixel size of 127 µm was used. Aiming at a sufficiently good sampling scheme during the measurement process, we used a spherical t-design of strength 13 with effectively 94 sampling directions (c.f. (2)). These directions are symmetric and consequently, due to the symmetry in our reconstruction problem, we only use unique directions up to mirroring. This yields 47 primary sensitivity directions. Each of these directions was measured from 45 views that form a tomographic set over 360° around the given axis.

Accounting for the geometrical restrictions of the rotation device, this amounts to 1404 viewpoints in total. Eight phase-stepping images with an exposure time of 2 s were recorded for all viewpoints over a total time period of approximately 11 hours.

For the dark-field images, the recorded raw data was binned by a factor of 4 prior to any processing, yielding an effective pixel resolution of isotropic 0.508 mm. This preprocessing step was only added to reduce the memory load during the reconstruction stage and does not impose a general limitation of the method. The absorption and the dark-field images are extracted using a cosine fitting according to (1, 3). For details of the setup we refer to (1, 3, 4, 5).

For the AXDT reconstruction we model the scattering using a field of spherical functions. Mathematically, this is a function $\eta(u,x)\colon \mathbb{S}^2\times\mathbb{R}^3 \rightarrow \mathbb{R}$ where x determines the position within the sample and u defines the scattering direction of interest ($\mathbb{S}^2 := \{u \in \mathbb{R}^3, |u| = 1\}$ denotes the surface of a unit sphere in 3D space). Further a weighting based on the detectability is introduced, $h(u,t,l)\colon \mathbb{S}^2\times\mathbb{S}^2\times\mathbb{S}^2 \rightarrow \mathbb{R}$. This function maps the scattering direction of interest u, the grating orientation t and the X-ray direction l to a single weighting factor as proposed by Malecki et al. (6). In a recent publication (5) we use real-valued spherical harmonics $\{V_k^m\}_{\{k\geq 0, -k\leq m\leq k\}}$, which constitute a basis for spherical functions, to describe the forward model. The simulation of a single anisotropic dark-field measurement d can thus be approximated as

$$d = \exp\left(-\frac{1}{4\pi}\sum_{k=0}^{K}\sum_{m=k}^{k} h_k^m(t,l)\int_L \eta_k^m(x)dx\right)$$

with L denoting the X-ray, $h_k^m(t,l)$ denoting the spherical harmonics coefficients of $h(u,t,l)$ and $\eta_k^m(x)$ denoting those of $\eta(u,x)$. This forward model enables the tomographic reconstruction of the scattering profile in each location of the specimen with respect to spherical harmonics.

The tomographic reconstruction of both the absorption as well as the AXDT (5) was carried out using our C++ framework CampRecon (7). For the X-ray projection model, we used a ray-driven multi-GPU projector developed by Fehringer et al. (8) written in OpenCL. In order to compute the tomography data set, we used the method of conjugate gradients (9) with 20 iterations. The machine, on which the tomography was computed, is equipped with dual Intel Xeon E5-2687W v2 with 128 GB RAM and dual Nvidia GeForce GTX 980Ti GPU accelerators. The AXDT reconstruction was performed according to (5), which reconstructs the scattering profile in each position of the specimen using spherical harmonics. The setup weighting function has been set to $h\colon \mathbb{S}^2\times\mathbb{S}^2\times\mathbb{S}^2 \rightarrow \mathbb{R}\colon (u,t,l) \mapsto (|l\times u|\langle u,t\rangle)^2$ according to Malecki et al. (6). This leads to a maximum degree of the spherical harmonics of $K = 4$ and degrees $k = 0,2,4$, as the scattering profile is assumed to be symmetric, effectively leading to 15 coefficient volumes η_k^m.

The tomographic reconstruction of the (unbinned) linear attenuation coefficients took 85 min, while the AXDT tomography took 18 min. Please note that the reduced time in case of AXDT is due to the reduced resolution. In general, the complexity of AXDT is 15 times higher than the one of the standard computed tomography.

In order to compute the Funk-Radon transform for a reconstructed scattering field $\eta(u,x)$ at location x, i.e.

$$\hat{\eta}(u,x) := \int_{C(u)} \eta(u',x)\, ds(u')$$

with $C(u) := \{u' \in \mathbb{S}^2,\ \langle u',u\rangle = 0\}$ denoting the great circle orthogonal to the direction u, we use the direct and highly efficient formula based on spherical harmonics. Meaning that the spherical harmonics coefficients $\hat{\eta}_k^m(x)$ of $\hat{\eta}(u,x)$ can be directly computed from the spherical harmonics coefficients $\eta_k^m(x)$ of the reconstruction $\eta(u,x)$ as (10):

$$\hat{\eta}_k^m(x) = P_k(0)\eta_k^m(x)$$

with P_k denoting the Legendre polynomials:

$$P_{2k+1}(0) = 0, \qquad P_{2k} = (-1)^k \frac{1 \cdot 3 \cdot 5 \cdots 2k-1}{2 \cdot 4 \cdot 6 \cdots 2k}$$

After this transform we consider peaks in $\hat{\eta}(u, x)$ to correspond to the fiber directions. For maxima detection, we used the method recently proposed in (11) with 1500 directions well distributed on the sphere computed by Voronoi tesselation (12, 13). The neighborhood is computed via the spherical Delauney triangulation (14).

The visualization of the fiber tracts in fig. 3 D) was created with the ImFusion Suite (15).

References and Notes:

1. Pfeiffer, F. *et al*. Hard-X-ray dark-field imaging using a grating interferometer. *Nat. Mater.* 7, 134–137 (2008).
2. Hardin, R. H. & Sloane, N. J. A. McLaren's improved snub cube and other new spherical designs in three dimensions. *Discrete Comput Geom* 15, 429–441 (1996).
3. Pfeiffer, F., Weitkamp, T., Bunk, O. & David, C. Phase retrieval and differential phase-contrast imaging with low-brilliance X-ray sources. *Nat. Phys.* 2, 258–261 (2006).
4. Donath, T. *et al*. Inverse geometry for grating-based x-ray phase-contrast imaging. *J. Appl. Phys.* 106, 054703 (2009).
5. Wieczorek, M., Schaff, F., Pfeiffer, F. & Lasser, T. Anisotropic X-Ray Dark-Field Tomography: A Continuous Model and its Discretization. *Phys. Rev. Lett.* 117, 158101 (2016).
6. Malecki, A. *et al*. X-ray tensor tomography. *EPL (Europhysics Letters)* 105, 38002 (2014).
7. Wieczorek, M., Vogel, J. & Lasser, T. CampRecon. Tech. Rep., Technische Universität München (2014).
8. Fehringer, A., Lasser, T., Zanette, I., Noël, P. B. & Pfeiffer, F. A versatile tomographic forward- and back-projection approach on multi-GPUs. In Ourselin, S. & Styner, M. A. (eds.) *SPIE Medical Imaging*, 90344F (SPIE, 2014).
9. Hestenes, M. R. & Stiefel, E. Methods of conjugate gradients for solving linear systems. *J. Res. Natl. Bur.* 49, 409 (1952).
10. Funk, P. Über Flächen mit lauter geschlossenen geodätischen Linien. *Math.Ann.*74,278–300 (1913).
11. Wieczorek, M., Pfeiffer, F. & Lasser, T. Micro-structure orientation extraction for Anisotropic X-Ray Dark-Field Tomography. In *Fully3D* (2017).
12. Du, Q., Faber, V. & Gunzburger, M. Centroidal Voronoi Tessellations: Applications and Algorithms. *SIAM Rev.* 41, 637–676 (2006).
13. Burkardt, J. SPHERE CVT . https://people.sc.fsu.edu/ ˜jburkardt/ m_src/sphere_cvt/sphere_cvt.html (accessed: 05.08.2018)
14. Burkardt, J. SPHERE DELAUNAY. https://people.sc.fsu.edu/ ˜jburkardt/m_src/sphere_delaunay/sphere_delaunay.html (accessed: 05.08.2018)
15. ImFusion GmbH, ImFusionSuite. https://www.imfusion.de (accessed: 05.08.2018)
